# Diencephalic Syndrome: Misleading Clinical Onset of Low-Grade Glioma

**DOI:** 10.3390/curroncol30090610

**Published:** 2023-09-13

**Authors:** Milena La Spina, Manuela Caruso, Carmela Gulizia, Mattia Comella, Rachele Soma, Mariaclaudia Meli, Piera Samperi, Gregoria Bertuna, Andrea Di Cataldo, Giovanna Russo

**Affiliations:** 1 Pediatric Hematology and Oncology Unit, Department of Clinical and Experimental Medicine, University of Catania, 95123 Catania, Italy; mlaspina@unict.it (M.L.S.); carmelagulizia@hotmail.it (C.G.); mattiacomella@gmail.com (M.C.); rachelesoma@gmail.com (R.S.); mclaudiameli@gmail.com (M.M.); psamperi@unict.it (P.S.); oriettabertuna40@gmail.com (G.B.); adicata@unict.it (A.D.C.); 2 Pediatric Endocrinology and Diabetology Center, Department of Clinical and Experimental Medicine, University of Catania, 95123 Catania, Italy; manuela.caruso@unict.it

**Keywords:** diencephalic syndrome, low-grade glioma, failure to thrive

## Abstract

**Simple Summary:**

Diencephalic Syndrome (DS) is a rare disorder secondary to an intracranial neoplasm typically located in the hypothalamic region or its vicinity. Lack of specific symptoms and latency of overt neurologic impairment are misleading and delay diagnosis. A multidisciplinary evaluation is essential to exclude the most frequent causes of failure to thrive, and it is essential to underline the need for pediatricians to focus their attention on this dangerous cause of poor weight gain or stunting in childhood in order to make an early diagnosis and improve prognosis and quality of life.

**Abstract:**

Background: Diencephalic Syndrome is an atypical early manifestation of low-grade gliomas; so, it is important to detect it in patients that experience a failure to thrive despite adequate length growth and food intake. The purpose of this article is to focus attention on this rare but potentially dangerous cause of poor weight gain or stunting in childhood. Materials and Methods: We describe four patients with Diencephalic Syndrome and low-grade gliomas who were evaluated in our institution from January 2017 to December 2021. Case Description and Results: two patients presented with suspected malabsorption, and two presented with a suspected eating disorder. In all cases, neurological symptoms appeared late, explaining the reason for the diagnostic delay, which impacts negatively on prognosis and on quality of life. Currently, patients 1 and 2 have stable disease in second-line therapy, patient 3 has stable disease post end of second-line therapy, and patient 4 has stable disease in first-line therapy. Everyone is in psychophysical rehabilitation. Conclusions: A multidisciplinary evaluation is essential in order to make an early diagnosis and improve prognosis and quality of life.

## 1. Introduction

Diencephalic Syndrome (DS), also known as Russell’s Syndrome, may be an atypical onset of low-grade gliomas (LGG) [1,2]. It is a very rare disorder, traditionally encompassing severe failure to thrive, emaciated body, poor weight gain/loss despite adequate length growth and food intake, nystagmus, and hyperkinesis, secondary to an intracranial neoplasm classically located in the hypothalamic region or its vicinity [1,2,3]. Since the first description in the 1930s, more than a hundred childhood cases have been published, considering either case series or single case reports [3,4,5,6]. The prevalence and incidence of the association between DS and LGG are not known because it is often misdiagnosed [7,8,9].

We report four patients with this association; in one of them, the tumor was not in the hypothalamus. Two of them had been referred to a general pediatric ward with suspected malabsorption, and two to a psychotherapist with a suspected eating disorder. In all cases, neurological symptoms appeared late, explaining the reason for the diagnostic delay. Diagnostic delay negatively impacts prognosis and quality of life, and for this reason, we wish to draw the attention of pediatricians to this rare but potentially dangerous cause of poor weight gain or stunting in childhood.

## 2. Materials and Methods

This is a retrospective monocentric study conducted from January 2017 to December 2022 that includes 4 patients with DS as an early manifestation of LGG. These patients are part of a cohort of 48 children with brain tumors diagnosed in the same period at our institution; 16/48 were low-grade gliomas, and 4 of them had signs/symptoms compatible with diencephalic syndrome at onset.

A literature review was performed on the main scientific electronic databases (PubMed, Embase, Google Scholar and Cochrane) by two authors (L.S.M. and G.C.) using the following keywords: children, pediatric and its variations; low grade glioma(s), pylocitic astrocytoma, chiasmatic/hypothalamic glioma; diencephalic syndrome, failure to thrive, poor weight, malabsorption, anorexia nervosa, eating disorders, neurobehavioral manifestations.

The literature search included only English articles that were published from 1952 to 2023. We selected potentially relevant articles according to titles and abstracts and examined the full-text articles for relevance and inclusion/exclusion. The articles had to have included pediatric patients ≤18 years of age at the time of tumor diagnosis; diagnosis of LGG and DS. Patients with high-grade malignant neoplasms and patients who experienced failure to thrive or neurobehavioral symptoms after diagnosis or because of treatment and patients whose signs/symptoms were potentially attributable to other conditions such as genetic/endocrinological/gastrointestinal/mental disorders were excluded. Children diagnosed with low-grade brain tumors and symptoms/signs at the onset compatible with Diencephalic Syndrome and were aged ≤18 years were included.

For each patient, the following data were collected by reviewing electronic medical records: age, sex, weight, height, body mass index (BMI), endocrinological assessment and IGF1 levels at diagnosis, medical history, cancer-specific history and related therapies.

## 3. Case Descriptions

### 3.1. Case 1

This patient was born at 38 weeks of gestation; birthweight was 3.285 kg and length was 50 cm (both parameters between the 25° and 50° centile). At 1.5 years of age, he had pallor, persistent cough and recurrent vomiting, normal linear growth, and normal head circumference, but poor weight gain despite adequate caloric intake and regular neurological developmental (historical data). He was admitted to the gastroenterology unit and screened for malabsorption diseases, endocrine disorders and allergies. Stool sample tests ruled out bacterial, fungal, and parasitic infections; fecal calprotectin, routine blood tests and nutritional values were normal. At the age of two, due to persistence of symptoms, he was admitted again. The 5-day food diary revealed an adequate caloric intake despite the low weight gain. Sweat chloride, celiac screen, thyroid function testing, immunoglobulins, endoscopic abdominal ultrasound, chest X-ray, ocular examination, esophageal pH-metry, esophagogastroduodenoscopy, phoniatric and swallowing tests and an arginine test to identify GH deficiency failed to reveal any diagnoses, except for evidence of pharyngoesophageal reflux, for which he received specific treatment with no benefit of weight gain.

At the age of 2.5 years, both height and weight were severely retarded (Table 1). At the age of 3.6 years, due to the persistence of symptoms even in the absence of neurological signs/symptoms, a brain and spinal MRI were performed, which revealed an exophytic and expansive neoformation in the bulbar region, measuring 13 × 10 × 11 mm. The diagnosis of tumor-related DS was made 2 years after the onset of symptoms and the first admission. Complete resection was not feasible, and the mass was only partially removed. Diagnosis was pilocytic astrocytoma (WHO G1), and immunohistochemical analyses were negative for BRAF V600E. Adjuvant therapy was administered for 18 months according to the International Society of Pediatrics Oncology (SIOP) protocol for low-grade glioma 2004 with a combination of vincristine and carboplatin.

Stop-therapy brain MRI confirmed the stable status of the neoplastic residual, and at stop-therapy, clinical examination showed good clinical conditions and satisfactory height and weight catch-up growth (Table 1).

Unfortunately, 18 months later, MRI showed a new increase in the volume of the residue, without clinical symptoms. It was decided that second-line therapy be started, consisting of 18 months of weekly vinblastine that he will complete in the next 2 months.

Currently, he has a stable disease, and both height and weight growth catch-up continue.

### 3.2. Case 2

This patient was born at 38 weeks of gestation. Birthweight was 3.420 kg, and length was 50 cm (both 50th percentile).

At the age of 0.4 years of age, exclusively breastfed, the infant had several episodes of regurgitation and vomiting, which led to a refusal to feed and a slowing of linear growth; there was minimal response to antireflux medications. The growth graph showed progressive weight loss, going from the 50th to the 25th percentile. There were no other specific signs or symptoms. Due to the persistence of symptoms when he was 6 months old, he was admitted for the first time to the pediatric gastroenterology unit. In spite of the poor growth and emaciated appearance, the child was lively and cheerful. Baseline investigations, including complete blood count, hepatic and renal function test, infectious screening with stool cultures, thyroid hormones, electrolytes, total protein, urine analysis, sweat chloride, abdominal ultrasound and heart control were normal.

At the age of ten months, seven months after the onset of symptoms and the first hospitalization, due to vertical nystagmus in the right eye, doctors prescribed a transfontanellar ultrasound. It identified “an ellipsoidal solid neoformation with rather demarcated margins, with a homogeneous echo structure” located in the hypothalamic/pituitary region. The child immediately underwent a brain and spinal MRI, which confirmed a voluminous mass measuring 49 × 35 × 37 mm, and he was transferred to our pediatric oncology unit. Latency between symptom onset and diagnosis was 7 months.

At the time of admission to the pediatric oncology unit, the child was very emaciated and had a dystrophic appearance with hypotrophic muscles and abdomen excavatum. (see Table 1 for auxologic data). Neurological examination revealed only the right eye nystagmus.

After multidisciplinary evaluation, a partial exeresis of the tumor was indicated, but an intraoperative hemorrhage required a more extensive resection. Post-surgical neurological examination showed left hemiparesis and a severe bilateral deficit of visual functions and acuity (worse in the left eye). The inconstant vertical nystagmus of the right eye was still evident, and the young patient also developed central palsy of the left facial nerve. Hormone screening reported low cortisol and ACTH levels; therefore, hydrocortisone therapy was started.

Histological diagnosis was grade I WHO pilocytic astrocytoma, and immunohistochemical analysis revealed no BRAF mutation. An intensive program of rehabilitation was started. A brain and spinal MRI 2 months after surgery showed an increase in residual tumor; therefore, adjuvant chemotherapy was started in accordance with the SIOP protocol for LGG, with a combination of vincristine and carboplatin for 18 months. A new brain and spinal MRI assessment 10 months after surgery, as well as 8 months later, confirmed stable disease. At stop-therapy, auxologic parameters showed stable height velocity and an increase in weight gain (Table 1).

Two years after the end of treatment, a brain MRI showed an increment in the size of the residual tumor, together with a worsening of the neurological symptoms in the absence of symptoms or signs of DS. Surgery was not an option due to the location of the tumor, so a second-line treatment with weekly vinblastine was performed for 18 months. Stop brain and spinal MRI showed stable disease. The known postsurgical neurological sequelae are still present. The left hemiparesis had some improvements thanks to physiotherapy, speech and psychomotor therapy, and his gait allows him to safely walk, although the mother reports episodic falls. During follow-up, he maintained stable height and weight growth, showing a stature not far from mean parental height SDS and constant excess weight (at Table 1).

### 3.3. Case 3

This patient was born at 39 weeks of gestation. Birthweight was 3.300 kg; length was 50.5 cm (both around the 50th percentile).

Her medical history starts at the age of 11 years with progressive weight loss, normal height and selective feeding. Many diagnostic investigations were conducted, particularly specific evaluations to rule out dysmetabolic syndromes, but the results were non-significant.

From the age of 15 years, she was intermittently under the care of a psychotherapist in the infantile neuropsychiatry unit of a suburban hospital because of an eating disorder characterized by food selectivity and restriction of caloric intake. The first diagnosis was anorexia nervosa. Pubertal development was not completed, and the patient was amenorrheic. Hypogonadotropic hypogonadism was documented.

After 1 year of specific and intensive psychological rehabilitation, inconstant headaches and progressive severe loss of weight occurred, followed eight months later by vomiting, photophobia, dysarthria and paresthesia.

Only after 4 years from the onset of symptoms (at the age of 15), due to the persistence of intense headache episodes with blurred vision and a partially altered state of mind, a brain MRI was performed that identified a voluminous mass measuring 20 × 34 × 30 in the suprasellar region, which occupied the anterior recess of the third ventricle and expanded both the lateral and third ventricles. The lesion also presented a strong contrast enhancement, and there was a significant leptomeningeal spread in both the brain and spinal cord. A stereotactic brain biopsy of a more superficial nodule was performed in the right cortical-subcortical temporal region with a definitive histological diagnosis of suprasellar pilocytic astrocytoma WHO grade I and with extensive leptomeningeal seeding in the brain and spinal cord and no BRAF V600E mutation. Physical examination at diagnosis reported a poor health condition and growth retardation (Table 1); she was very asthenic and had abundant subcutaneous tissue in the abdomen, muscular hypotrophy, good muscular strength in the four limbs, intact sensorium and speech, apparently intact cranial nerves, diffuse tremors and inability to walk independently. In addition, she had diffuse pain not responsive to analgesic therapy. Adjuvant chemotherapy, according to the SIOP protocol for LGG, with a combination of vincristine and carboplatin for 18 months was started.

At the end of induction chemotherapy, no height catch up was observed, but further weight loss occurred (Table 1). During treatment, several MRI assessments documented a stable disease with an improved clinical-neurological picture, autonomous walking, and absence of pain, but persisting poor weight gain with low caloric intake. About six months after stop-therapy, and due to the persistent episodes of cephalalgia, emesis and dizziness, second-line chemotherapy was started with a weekly dose of vinblastine, which was poorly tolerated. Therefore, proton therapy was discussed, and new treatment started. However, during the 12 months following the stop therapy, a significant catch-up in weight gain was observed (Table 1). MRI examination three months after proton therapy was stable, and the same exam six months later documented for the first time a reduction in the size of the main neoplastic lesion in the hypothalamic/pituitary region (Ø 10.5 × 41 mm vs. Ø 21 × 40 mm), and the known leptomeningeal dissemination was confirmed as stable. In addition, increased appetite, gain weight and mood stabilization were observed. The most recent MRI evaluation one year after proton therapy did not show any signs of disease progression. To date, the patient is in replacement therapy for hypogonadotropic hypogonadism and presents as spastic-ataxic, but she has an autonomous gait and no tremors, memory impairment or difficulty concentrating. Further weight gain occurred with improved BMI (Table 1). The Wechsler Adult Intelligence Scale (WAIS-IV) test describes below-average cognitive abilities (IQ 78). She practices intense periods of neuropsychophysical rehabilitation associated with pet therapy and dance therapy.

### 3.4. Case 4

The patient was born at 39 weeks of gestation. Birthweight was 2.840 kg; length was 48 cm (both parameters around the 25th percentile). Her medical history started at the age of 10 years with mood and eating disorders characterized by food selectivity and restriction of caloric intake, progressive weight loss and a slowing of her linear growth rate. Also, in this case, the first diagnosis was an eating disorder with an indication for psychological support. She was admitted at the infantile neuropsychiatry unit, where several tests were performed, including an electroencephalogram that highlighted diffuse non-specific anomalies. Therefore, after about twelve months, a brain MRI was performed that showed evidence of a “left paramedian suprasellar expansive hypothalamic lesion” measuring 20 × 18 × 32 mm. Latency between symptoms onset and diagnosis was one year. The young patient was then hospitalized at the neurosurgery unit, and the mass was biopsied. The histopathology report described a diffuse suprasellar pilocytic astrocytoma (WHO G1), and immunohistochemical analyses were negative for BRAF V600E.

The physical examination reported inadequate height and weight; her skin appeared pale pink, while her subcutaneous fat and muscular masses were poorly evident. Chemotherapy following the SIOP protocol LGG 2004 was started.

During therapy, the patient experienced difficult-to-interpret bone pain associated with behavioral disturbances; neuropathic pain due to vincristine toxicity could not be ruled out with certainty, so the dose was reduced by 25% for the entire subsequent period, and the treatment was discontinued 6 months earlier at the behest of the parents.

To date, five months after stop-chemotherapy, an MRI showed stable disease, and auxological parameters showed persisting slow height velocity and moderate weight gain (Table 1).

## 4. Discussion

Pediatric LGGs are the most common type of central nervous system (CNS) tumors in children; they occur preferentially during the first decade of life, tend to stabilize after puberty, and develop in 15–20% of children with neurofibromatosis type 1 (NF1) [10,11]. Around 30–50% typically arise from the optic pathway and involve the hypothalamus and the diencephalic structures [7,8,9].

Diencephalic Syndrome (DS) may be an atypical onset of low-grade glioma (LGG). It was first described in the 1930s and, later, better characterized by Russel in 1951 [1,2]. Since the first description, more than a hundred childhood cases have been published when considering either case series or single case reports [12,13,14,15,16]. Unfortunately, the prevalence and the incidence of the syndrome are not known probably because the condition is largely underdiagnosed [6,7].

DS is usually associated with LGG, typically arising from the optic pathway and involving the hypothalamus and the diencephalic structures; cases associated with craniopharyngioma or brain stem neoformations have been reported, although they are rare. Approximately 9% of tumors are located elsewhere, including the posterior fossa and anterior hypothalamus. Current evidence suggests that a rare population of children with a similar phenotype may have their tumor located in the posterior fossa, explaining the DS-like presentation, a rare entity with few cases reported in the literature [17,18,19].

The majority of cases are pilocytic astrocytoma (World Health Organization (WHO) grade I), with a smaller proportion being pilomyxoid astrocytomas and gangliogliomas (WHO grade II) [4,7,8,9].

DS is a very rare disorder characterized by a severely emaciated body and poor weight gain or weight loss despite food intake. Linear growth is usually maintained, and there is normal or precocious intellectual development, hyperalertness, and hyperkinesis; typically, the little patients often have a cheerful outgoing manner, which is in contrasts to their outward appearance. Other case series have reported nystagmus, visual field defects, skin pallor, hypotension, hypoglycemia, headache, and emesis [18].

In recent reports, the importance of brain tumors presenting as psychiatric conditions was emphasized; a major finding was the prevalence of hypothalamic tumors in patients with anorexia nervosa, prompting the authors to recommend neuroimaging for such patients [20,21,22].

Considering that these symptoms are not specific of central nervous system (CNS) tumors but have much in common with other pediatric diseases, and given that neurological symptoms appear late, a diagnostic delay is frequent and risks the growth of the neoplasm, which worsens the disease-free survival and quality of life [9,23,24].

The etiopathogenesis of DS is still under study. There is growing interest in the role of GH; it has been noted that some patients had elevated baseline GH levels and none had appropriate GH suppression after glucose loading. The increase in GH levels could be secondary to the release of GH-releasing factors from the hypothalamus and could result in lipolytic activity underlying the absence of subcutaneous fat observed in children with DS [25,26,27,28,29].

We analyzed the clinical features, timing to diagnosis, site of onset, management and outcomes of four cases of low-grade glioma and Diencephalic Syndrome diagnosed in our institution from January 2017 to December 2022. These patients were part of a cohort of 48 children with brain tumors diagnosed in the same period at our institution; 16/48 were low-grade gliomas, and four of them had signs/symptoms compatible with diencephalic syndrome at onset.

In two of the four patients analyzed, the tumor was diencephalic; one was diencephalic with extensive leptomeningeal seeding in brain and spinal cord, and one was in an atypical bulbar site.

At the onset of symptoms, two of them (cases 1–2) were referred to general pediatric ward with a suspicion of malabsorption, and the other two (cases 3–4) were referred to a psychotherapist in an infantile neuropsychiatry unit with a suspected eating and behavior disorder.

In all our cases, there was a diagnostic delay, with a mean latency of 22.75 months (range 7–48) between symptom onset and diagnosis, in agreement with the data published in the literature. This diagnostic delay was not found in the other twelve LGGs diagnosed in the same period at our department.

All four patients underwent a surgical biopsy, and after histological diagnosis, all of them received upfront chemotherapy in accordance with the SIOP LGG 2004 protocol and supportive care.

It has been reported that astrocytoma associated with DS tend to be larger, more aggressive, and occurs at a younger age than those without DS. Spinal seeding is associated with poor prognosis, emphasizing the importance of early diagnosis [6].

Currently, all our cases are alive, although three of them experienced tumor progression. None were eligible for treatment with therapy-targeting BRAF. Patient 1 has stable disease and is in second-line therapy with vinblastine, patient 2 has stable disease and is 2 months post second-line therapy with vinblastine, patient 3 has stable disease and is 22 months post second-line therapy with vinblastine and proton-therapy, and patient 4 has stable disease and is 6 months post first-line therapy. Long-term sequelae included visual, endocrinological and neuropsychological dysfunction emphasizing how the precocious multidisciplinary approach is of great importance.

Each of them is followed by careful clinical, auxological, hematological and radiological monitoring, and everyone is in intensive psychophysical rehabilitation. In particular, from the psychological evaluations of cases 3 and 4 (which were initially misdiagnosed as eating and behavior disorders), a persistent altered relationship with food emerged; thus, we cannot exclude the possibility that DS and the eating disorder in cases 3 and 4, both female and adolescent, could coexist.

Case 1 is another case of a rare association of DS with an extrahypothalamic tumor. Current evidence suggests that a rare population of children may have their tumor located in the posterior fossa, explaining the DS-like presentation, a rare entity with few cases reported. In particular, our case with a bulbar tumor is reminiscent of another single case published in 2016 [18,19]. Both arose in the posterior fossa and present an unusual association between persistent cough and failure to thrive at onset. A hypothesis to explain the clinical overlap of the aforementioned symptoms is that posterior fossa tumors may interfere with the locus coeruleus and fourth ventricle, from where the hypothalamus receives significant neuronal input, interfering with the central respiratory drive through the involvement of the vagus or recurrent laryngeal nerves [19].

Retrospectively, in relation to the emerging pathogenetic hypotheses on the correlation between GH value and DS, we examined the endocrinological screening at disease diagnosis. IGF1 levels were measured, and all values were in the normal range for their age.

Case 1: IGF1 62.6 ng/mL (n.v. 17–347 ng/mL)

Case 2: IGF1 34.7 ng/mL (n.v. 17–347 ng/mL)

Case 3: IGF1 180 ng/mL (n.v. 17–347 ng/mL)

Case 4: IGF1 195 ng/mL (n.v. 17–347 ng/mL)

In our case series at disease diagnosis, three out of the four patients (cases 1, 3, 4) exhibited both a height and weight deficit, although the weight deficit was more severe than the height deficit; case 2 was the youngest, and despite the delay in diagnosis, the time from onset of the disease was probably not sufficient to cause a deflection of height below normal limits. Both cases 1 and 2 exhibited good catch-up growth, especially for weight, after start of therapy; during follow-up, height growth also normalized. In cases 3 and 4, disease onset occurred during pubertal age and, as stated, probably coexisted with an eating disorder; height and weight growth were impaired both before and after diagnosis because of the summative effect of DS, absence of pubertal growth spurt and inadequate nutrition; however, after therapy and/or during follow-up, some weight gain was also observed in these two girls, and in case 4, the first sign of puberty appeared.

DS is a frequently neglected cause of failure to thrive in infants and children, and the availability of reports may improve the medical awareness of this condition. The authors want to stress the importance of diagnostic delay both for prognosis and for quality of life, underlining the need to focus the attention of pediatricians, child psychologists and neuropsychiatrists on DS, a rare but potentially dangerous cause of poor weight gain or stunting, so that they can plan careful clinical-neurological monitoring and carry out a complete eye examination, neuroendocrine evaluation and possibly a brain magnetic resonance when the symptoms persist without diagnosis.

In addition, it is important to establish clear criteria for neuroimaging indications in children and adolescents with eating disorders.

## 5. Conclusions

DS is a rare condition with a changeling diagnosis that often poses a difficult challenge for pediatricians. In all our cases with diagnostic delay, a mean latency of 22.75 months (range 7–48) between symptom onset and diagnosis was consistent with the data published in the literature.

It is imperative that a multidisciplinary evaluation be conducted with a complete gastroenterological, endocrinological and neurological workup to exclude the main causes of malabsorption and failure to thrive and reach an early diagnosis to improve prognosis and quality of life, without forgetting that even eating disorders can sometimes be indicated by a neuroimaging study.

## Figures and Tables

**Table 1:** Auxological data of cases.

Case 1	C.A.	Height cm	Height SDS	Weight kg	Weight SDS	BMI	BMI SDS
	2.6	84	−2.2	9.9	−3.4	13.9	−2.1
Diagnosis/Start therapy	3.6	91	−2.2	11	−3.6	13.3	−2.5
End induction	4.1	95	−2.0	13.9	−1.9	15.3	0.3
Stop therapy	5.1	104	−1.5	17	−1.1	15.7	−0.07
Follow-up	8.6	128	−0.6	25.5	−0.9	15.5	−0.8
Target height			0.5				
Case 2	C.A.	Height cm	Height SDS	Weight kg	Weight SDS	BMI	BMI SDS
Diagnosis/surgery	0.9	72	−0.3	7	−2.3	13.5	−3.0
Start Therapy	1.0	73	−0.9	8	−1.5	15	−1.5
End induction	1.5	79	−0.9	10.7	−0.05	17.1	0.7
Stop therapy	2.4	88	−1.0	14.3	0.2	18.4	1.2
Follow-up	4.9	104	−1.0	22.8	1.0	21	1.9
Follow-up	6.9	116	−1.1	26	0.3	19.3	1.2
Target height			−0.7				
Case 3	C.A.	Height cm	Height SDS	Weight kg	Weight SDS	BMI	BMI SDS
Diagnosis/Start therapy	15.8	150	−1.9	33.7	−3.7	14.9	−3.3
End induction	16.2	150	−2.0	31.3	−4.5	13.9	−4.4
Stop therapy	17.3	150	−2.0	30	−5.0	13.3	−5.3
Proton therapy	18.2	150	−2.1	33	−4.2	14.6	−3.9
Follow-up	19.4	150	−2.1	36	−3.4	16	−2.7
Follow-up	21.1	150	−2.1	42	−2.5	18.2	−1.5
Target height		153	−0.7				
Case 4	C.A	Height cm	Height SDS	Weight kg	Weight SDS	BMI	BMI SDS
Diagnosis/Start therapy	10.9	124	−3.0	19	−3.8	12.3	−3.4
End induction	11.4	124.5	−3.4	19.5	−3.9	12.6	−3.3
Stop therapy	12.2	131	−3.5	22.5	−3.6	13.7	−2.7
Follow-up	12.5	135	−3.5	25	−3.3	13.7	−2.2
Target height		155	−1.3				

Height, weight and BMI were normalized for chronological age by conversion to SD scores using SIEDP software “Growth Calculator 4”, according to WHO 2006 references for data of children below 2 years and the Italian Cacciari’s Growth Charts for children above 2 years. Legenda: C.A. = Chronological Age.

## Data Availability

The data presented in this study are available in this article.
